# New and Emerging Targeted Therapies for Hidradenitis Suppurativa

**DOI:** 10.3390/ijms23073753

**Published:** 2022-03-29

**Authors:** Adela Markota Čagalj, Branka Marinović, Zrinka Bukvić Mokos

**Affiliations:** 1 Department of Dermatology and Venereology, University Hospital Centre Split, Spinčićeva 1, 21000 Split, Croatia; adela.markota@gmail.com; 2 School of Medicine, University of Split, Šoltanska 2, 21000 Split, Croatia; 3 School of Medicine, University of Zagreb, Šalata 3, 10000 Zagreb, Croatia; branka.marinovic@kbc-zagreb.hr; 4 Department of Dermatology and Venereology, European Reference Network (ERN), Skin Reference Centre, University Hospital Centre Zagreb, Kišpatićeva 12, 10000 Zagreb, Croatia

**Keywords:** hidradenitis suppurativa, biologics, TNF-α inhibitors, IL-17 inhibitors, IL-1 inhibitor

## Abstract

Hidradenitis suppurativa (HS) is a chronic, recurrent, inflammatory skin disease deriving from the hair follicles. The formation of inflammatory nodules, abscesses, fistulas, and sinus tracts is characterized by a large inflow of key pro-inflammatory mediators, such as IFN-γ, TNF-α, IL-1, IL-17, and IL-12/23. Adalimumab is currently the only Food and Drug Administration (FDA)- and European Medicines Agency (EMA)-approved biologic therapy for moderate to severe HS in adults and adolescents. However, the long-term effectiveness of this TNF-α inhibitor in HS patients has shown to be highly variable. This review aims to review the evidence for emerging therapies that target the main pro-inflammatory cytokines in HS pathogenesis. A review of the literature was conducted, using the PubMed and Google Scholar repositories, as well as Clinicaltrials.gov. Presently, the most promising biologics in phase III trials are anti-IL-17 antibodies, secukinumab, and bimekizumab. Furthermore, an anti-IL-1 biologic, bermekimab, is currently in phase II trials, and shows encouraging results. Overall, the clinical efficacies of all new targeted therapies published up to this point are limited. More studies need to be performed to clarify the precise molecular pathology, and assess the efficacy of biological therapies for HS.

## 1. Introduction

Hidradenitis suppurativa (HS) is a chronic, recurrent, inflammatory skin disease deriving from the hair follicles, affecting intertriginous skin, and commonly associated with various systemic comorbidities [1]. Due to the differences in populations and methodologies, the estimated prevalence of HS varies from 1–4% in numerous published papers. However, the prevalence rate of 1% has been widely accepted [2]. The disease is three times more common in women than men, and most frequently manifests itself between the ages of 18 and 29 [3]. HS is a multifactorial disease resulting from a combination of genetic, environmental, and immunologic factors. Primarily, it is associated with follicular occlusion, which can originate from many biological processes, including follicular hyperplasia and hyperkeratinization [4].

The clinical course is very variable, and ranges from mild clinical presentation, characterized by the formation of papules, pustules, and nodules, to severe cases of deep abscesses, draining sinus tracts, and keloid scars [5]. The affected sites most commonly include intertriginous areas, particularly the axillae and anogenital region in both genders, and the submammary folds in women. Uncommon affected sites include the areola of the breast, the periumbilical skin, the scalp, the zygomatic and malar areas of the face, the buttocks, the thighs, and the popliteal fossa [6]. Because of the severe pain, pruritus, malodorous discharge, sleep, sexual dysfunction, and low self-esteem, HS has a notable impact on a patient’s quality of life and professional activity [7].

HS has commonly been staged according to the Hurley staging system (Table 1), a useful tool for determining the severity of the disease in individual patients, but it is deficient for assessing therapeutic response in clinical trials. Other used severity assessment scores are Hidradenitis Suppurativa Clinical Response (HiSCR), Hidradenitis Suppurativa–Physician Global Assessment (HS-PGA), Hidradenitis Suppurativa Severity Index (HSSI), Modified Sartorius Score (mSS), International Hidradenitis Suppurativa Severity Score System (IHS4) (Table 2), Dermatology Quality of Life Index (DLQI), Visual Analogue Scale (VAS) of pain, and Patient’s Global Assessment of Skin Pain (PGA-SP) [8,9]. Patients who achieve HiSCR should show a 50% reduction in the total inflammatory nodule count, with no increases in abscesses count and draining fistulas from the baseline. It is the most commonly used tool in research trials when assessing the efficacy of biologics [10].

Conventional medical therapy, involving topical treatment and oral antibiotics, and lifestyle modifications, including weight loss and cigarette smoking cessation, are suitable for treating mild to moderate HS [12]. With the discovery of the key pro-inflammatory cytokines involved in HS pathogenesis, the efficacy of biologics and other targeted therapies in the treatment of moderate to severe HS has been intensively evaluated [13].

This review aims to provide current insights into the underlying molecular pathophysiology of HS, and the evidence for new and emerging therapies that specifically target cytokines involved in HS pathogenesis.

## 2. Pathogenesis

The pathogenesis of HS is multifactorial, with genetic, environmental, and immunologic factors included in both the onset and maintenance of the condition [4].

### 2.1. Genetic Factors

Up to 42% of patients with HS report a family history of the condition [14]. The genetic mutations related to HS that have been published to date suggest that HS can be inherited as a monogenic disease, due to a defect in γ-secretase/Notch signaling pathway and inflammasome function, or more often as a polygenic disease resulting from defects in genes regulating proliferation of the follicular epithelium or immune system function [15].

γ-secretase is an intramembrane protease complex consisting of four hydrophobic proteins, presenilin (PSEN), presenilin enhancer-2 (PSENEN), nicastrin (NCSTN), and anterior pharynx defective 1 (APH1), which are encoded by *PSEN1/PSEN2, PSENEN, NCSTN*, and *APH1A/APH1B*, respectively [16]. It regulates canonical Notch signaling, which, amongst many processes, is involved in keratinocyte maturation and differentiation [17]. Notch signaling is enabled when Notch receptor (Notch1–4) contacts ligand (Jagged1, Jagged2, or one of three Delta-like proteins, DLL-1, -3 or, -4) on a neighboring cell. The intramembrane cleavage (S3-cleavage) of Notch is performed by γ-secretase, which releases Notch intracellular domain (NICD), which exhibits signaling activity in the nucleus. NICD enters the nucleus, associates with a DNA-binding protein CSL (RBP-J), and assembles a transcriptional complex [17]. Mutations of either the *NCSTN*, *PSEN1*, or *PSENEN* genes lead to an impaired γ-secretase activity with a deficient Notch signaling [17]. This results in hyperkeratinization of the follicular epithelium, and the formation of a keratin plug [4].

Recently, cases of syndromes associated with HS involving Notch pathway down-regulation have been published. *PSENEN* gene mutations were reported in patients with concomitant HS and Dowling-Degos disease [18], an autosomal dominant disorder characterized by acquired reticular hyperpigmentation in flexural sites.

It was reported that the Notch pathway could play a role in the human respiratory epithelium function, and that down-regulation of the Notch pathway gene expression is associated with smoking [19]. Also, Notch signaling has recently been emphasized as an important regulator of metabolism and metabolic syndrome [20]. Furthermore, it has been suggested that hyperandrogenism and polycystic ovarian syndrome play a role in the disease pathogenesis in female HS patients [21]. The precise connection between HS and its concomitant comorbidities is not fully confirmed, although the down-regulation in Notch signaling may present a reasonable explanation. To enlighten the exact pathogenic link, further studies are necessary.

An inflammasome is a cytoplasmic protein primarily located in macrophages, and is an important component of the innate immune system. It consists of a protein of the NOD-like receptor family (NLRP3), which senses microbial or damage products, and an enzyme, caspase-1, which proteolytically cleaves pro-IL1β into its active form, IL1β [22,23]. The important function of NLR molecules and IL-1β in autoinflammatory disorders has been recognized. One of them is Familial Mediterranean Fever, caused by a Mediterranean fever gene (*MEFV*) mutation, and reported in a series of patients to be concomitant with HS [24,25]. The *PSTPIP1* gene mutations, affecting proteins of the inflammasome, have been proven in PAPASH (pyoderma gangrenosum, acne, arthritis, and HS) [26], and PASH (pyoderma gangrenosum, acne, suppurative hidradenitis) syndromes [27].

### 2.2. Environmental Factors

Mechanical stress [28], metabolic syndrome [29], obesity [30], diabetes mellitus [31], diet [32], smoking [33], cutaneous microbiome [34], and hormonal factors [35] are involved in both the development and the maintenance of HS. Of note, evidence in the literature for cardiovascular (hypertension), endocrine (thyroid disorders, polycystic ovarian syndrome), gastrointestinal (inflammatory bowel disease), and rheumatologic comorbidities (arthropathies), as well as malignancy, is increasing [36]. Because of the effect of HS on general wellbeing, pain, self-esteem, sleep, and sexual dysfunction, clinicians involved in the care of patients with HS should be aware of the possible development of depression and suicidal thoughts in this population [37].

### 2.3. Immunologic Factors

**Figure 1:**
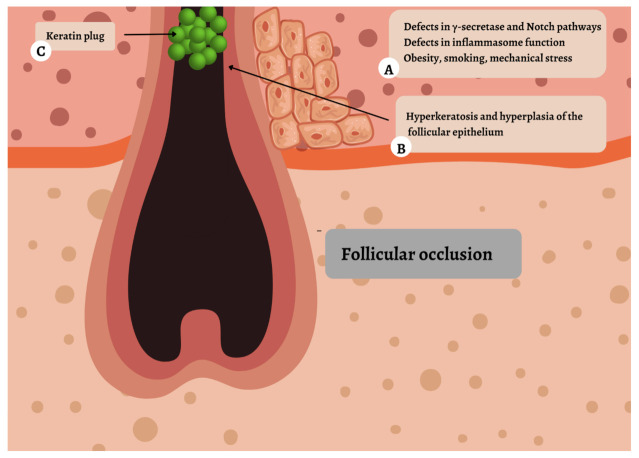
The first event in HS development is follicular occlusion. Genetic predisposition, mechanical stress, and environmental factors (A) induce hyperkeratosis and hyperplasia of the follicular epithelium (B). This results in the accumulation of cellular debris and the formation of a keratin plug (C).

Histopathological observations in very early HS lesions suggest that the primary event in the HS development is follicular occlusion (Figure 1) [38]. Follicular occlusion results from hyperkeratosis and hyperplasia of the follicular epithelium, accumulation of cellular debris, and the formation of a keratin plug [38].

**Figure 2:**
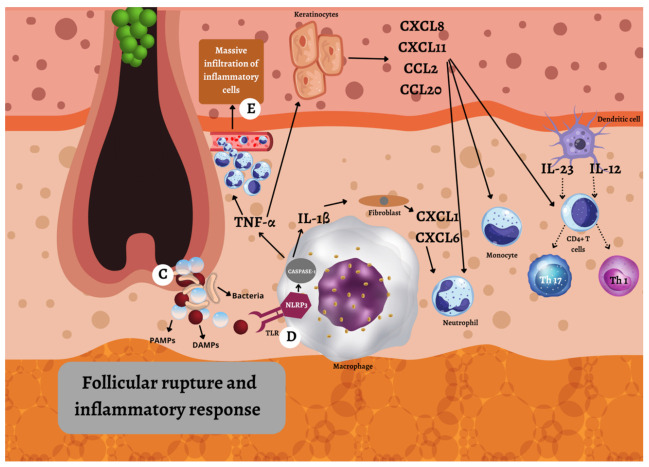
The second event is the rupture of the dilated follicle (C), and dispersing the keratin fibers, commensal flora, and PAMPs/DAMPs into the dermis. PAMPs and DAMPs are recognized by macrophages through TLRs and inflammasomes (D). Macrophages are, through TLRs, stimulated to produce TNF-α. The inflammasome is activated through an NLRP3 that senses microbial or damage products. It then mediates activation of caspase-1, which proteolytically cleaves pro-IL1-β into its active form, IL-1β. IL-1β activates fibroblasts which produce CXCL1 and CXCL6, and TNF-α activates keratinocytes which produce CXCL8, CXCL11, CCL2, and CCL20. These chemokines recruit more inflammatory cells, mainly neutrophils, monocytes, and subsets of T cells. Massive infiltration of inflammatory cells leads to nodules, abscesses, and fistula formation (E). Activated dendritic cells produce IL-12, which induces Th1 polarization and IL-23, which is responsible for maintenance of the Th17 phenotype.

The second event is rupture of the dilated follicle (Figure 2), and dispersing the keratin fibers, bacteria, and pathogen- and damage-associated molecular patterns (PAMPs/DAMPs) into the dermis [38]. This triggers an acute and severe immune response [38]. The enhanced mechanical friction at intertriginous body areas facilitates the rupture of the follicle, and disperses the follicle content into the dermis [38]. This is characterized by a large inflow of monocytes, neutrophils, multinucleated giant cells, B-cells, plasma cells, T-cells, and natural killer cells, leading to nodule and abscesses formation [38]. Toll-like receptors (TLRs) and inflammasomes located on/in the macrophages recognize PAMPs and DAMPs [39]. Macrophages are, through TLRs, stimulated to produce TNF-α [39]. The inflammasome is activated through NLRP3, which senses microbial or damage products. It then mediates activation of caspase-1, which proteolytically cleaves pro-IL-1β into its active form, IL-1β [22,23]. IL-1β activates fibroblasts which produce CXCL1 and CXCL6, and TNF-α activates keratinocytes which produce CXCL8, CXCL11, CCL2, and CCL20. These chemokines recruit more inflammatory cells [39].

The complement dysregulation is observed in HS, but it is uncertain whether elevated C5a and decreased C3b in serum of HS patients reflect a primary event in the HS pathogenesis, or result from advanced-stage disease. The primary function of C5 and C3 includes activation of the immune system via anaphylatoxins, opsonization, and bacterial lysis, all early events in HS. Elevated C5a in HS suggests complement hyperactivation, whereas decreased C3 results from using C3b for bacterial opsonization [40]. Clinical trials have shown that targeting C5a in the treatment of HS results in a high response rate [41].

In the Hurley stage II/III, nodules, abscesses, and fistulas are further formed, characterized by a massive infiltration of inflammatory cells and pro-inflammatory cytokines, including IFN-γ, TNF-α, IL-1, IL-17, and IL-12/23 [38]. Activated dendritic cells produce, amongst other effector molecules, IL-12 and IL-23, heterodimeric cytokines that share a common subunit (p40) [42]. IL-12 induces differentiating of naive CD4+ T cells to Th1. Th1 cells secrete TNF-α, lymphotoxin, IL-2, and IFN-γ. IFN-γ is a cytokine that facilitates the recruitment of circulating leukocytes, activates dendritic cells, and induces further Th1 differentiation [43]. It was found to be elevated in the wound exudate of HS patients [44]. Macrophages, activated dendritic cells, and T lymphocytes produce TNF-α [45]. There are several roles of TNF-α in the pathogenesis of HS. First, it supports Th17 polarization, increasing the ratio of Th17 to regulatory T-cells, which results in the increased production of HS-relevant cytokines [45]. Second, TNF-α suppresses the adipocyte secretion of adiponectin, an anti-inflammatory hormone that regulates glucose metabolism and insulin sensitivity. Adiponectin levels are significantly decreased in HS patients, which, accordingly, often have higher fasting serum glucose, insulin levels, and insulin resistance [45]. Third, the relationship between smoking and HS possibly involves TNF-α. Nicotine increases eccrine gland secretion, and its presence in sweat induces keratinocytes and Th17 cells to release TNF-α. Nicotine also directly stimulates macrophages to produce IL-1β and TNF-α [45]. Fourth, TNF-α increases the expression of TLRs and matrix metalloproteinases (MMPs) [45]. Other signaling molecules that interact with TNF-α have been found, i.e., Mammalian Target of Rapamycin (mTOR) [45]. The increased expression of mTOR has been reported in lesional and non-lesional skin of HS patients, and has been directly correlated with the disease severity [46]. The mTOR activation stimulates steroidogenic secretion, and promotes follicular adhesion with subsequent follicular plugging [46]. One study showed that adalimumab therapy strongly reduced mTOR1 expression [47]. IL-6 and TGF-β initiate differentiating of naive CD4+ T cells to Th17, whereas IL-23, IL-21, and IL-1β are factors responsible for maintenance of the Th17 phenotype [48]. The Th17 cells produce, amongst other cytokines, IL-17, which is elevated in HS [49], stimulating neutrophils and macrophages to produce more inflammatory cytokines, such as IL-1β, IL-6, and TNF-α, as well as caspases and MMPs [50]. IL-1β is a pyrogen and leukocyte-activating factor that further increases Th17 cell levels [50]. TNF-α, IL-17A, and IL-17F induce keratinocyte proliferation and production of IL-17C and IL-36, which act in an autocrine manner to further potentiate keratinocyte activation [39].

**Figure 3:**
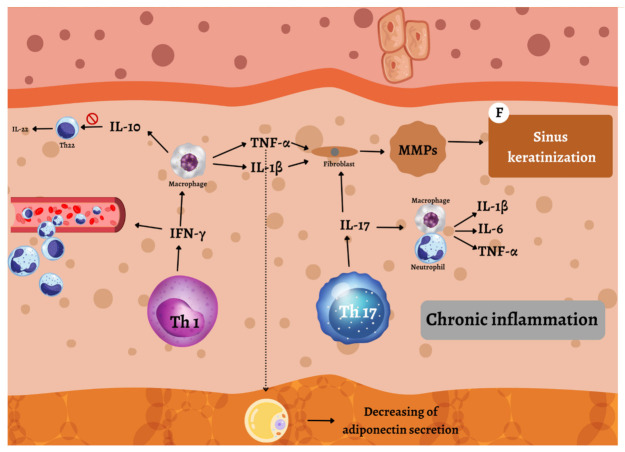
The third event in HS pathogenesis is chronic inflammation with sinus tract formation. Th1 cells produce IFN-γ, which recruits more inflammatory cells. Macrophages, activated dendritic cells, and T lymphocytes produce TNF-α, which has a multifactorial role. TNF-α supports Th17 polarization, suppresses adiponectin secretion, and induces the production of MMPs. Th17 cells produce IL-17 which stimulates neutrophils and macrophages to produce IL-1β, IL-6, TNF-α, and MMPs. TNF-α, IL-17, and IL-1β induce fibroblasts to produce MMPs, which lead to fibrosis and sinus tract formation (F).

After follicular rupture, dispersed follicular content and the cytokine-driven feedback propagate and sustain chronic inflammation, the third event in the HS pathogenesis (Figure 3) [38]. Increased MMPs and tissue inhibitors of metalloproteinases (TIMPs), together with the increased activity of fibrotic factors such as a tissue growth factor (TGF)-ß, result in the formation of epithelialized tunnels, sinus tracts, and keloid scars [38]. Epithelialized tunnels and sinus tracts provide an excellent habitat for the biofilm-producing bacteria, which continuously trigger inflammation, and are immunologically active [38]. Probably, circulating pro-inflammatory cytokines and chemokines from chronic lesions may activate the immune system of the hair follicle in distant predilection sites [38].

Despite many attempts to clarify the HS pathogenesis, it remains still poorly understood. Literature data describes the lack of antigen-specificity of T-cells involved in underlying pathophysiological mechanisms of HS, suggesting that HS is rather a cytokine-driven inflammatory process [51]. Recently, it has been recognized that bystander T cell activation plays an emerging role in inflammatory diseases, such as HS [52]. Bystander T CD4+ cell activation occurs when T cells are stimulated by inflammatory mediators such as cytokines or TLR signaling molecules (microbial and damage products) in the absence of antigen-specific T cell receptor (TCR) stimulation [52]. Subsequent to chemotaxis to the inflammatory environment, CD4+ T cells undergo differentiation to Th1 or Th17 cells, activating the two most prominent pathways in HS, followed by massive infiltration of inflammatory cells and pro-inflammatory cytokines [51]. Trials with immunomodulatory therapies support the role of altered cytokine signaling and dysregulated innate immune pathways, such as TNF-a, IL-1β, IL-12, IL-17, IL-23, and complement [51]. The targeted binding of biologics to one or two molecules helps elucidate the roles of different inflammatory pathways in HS pathogenesis based on different clinical responses to treatment when used empirically [51]. Various small molecule drugs that regulate inflammatory mediators are currently being investigated for HS (i.e., apremilast, Janus Kinase (JAK) inhibitors) [51].

## 3. Treatment

Therapy for HS includes topical, systemic, surgical, and combined treatment. General measures and additional medical treatments are recommended regardless of the disease severity [53].

### 3.1. Topical Therapy

The initial management of patients in Hurley stage I or mild HS usually includes a clindamycin 1% solution applied b.i.d. (lat. bis in die, twice a day) during 3 months in the skin areas subject to recurrent flares [53,54]. Patients who do not achieve disease control after 3 months of topical clindamycin usage are candidates for oral tetracyclines [53,54]. However, one study that compared the efficacy of topical clindamycin and systemic tetracyclines in HS patients during a minimum of 3-month follow-up showed no significant differences between the two therapies [55].

Resorcinol is a topical peeling agent with keratolytic, antipruritic, and anti-inflammatory properties that can be administered for HS (Hurley I or II), where available [53]. Patients are advised to apply resorcinol 15% cream b.i.d. directly to a new inflamed nodule [53]. The treatment should start as soon as possible after the patient perceives the beginning of a flare, preferably within several hours of symptom onset [53].

An intralesional corticosteroid injection involves the injection of a corticosteroid, such as triamcinolone acetonide, at a concentration of 5 to 10 mg/mL, directly into an inflammatory nodule [53]. A noticeable improvement in symptoms often occurs within 48–72 h. It is administered for HS patients with solitary abscesses, nodules, or sinus tracts resistant to other therapies [53].

A phase II open-label study (NCT04414514) assessing the efficacy of topical Janus Kinase (JAK) inhibitor (ruxolitinib 1.5% cream) in HS patients awaits recruitment of participants [56]. 

### 3.2. Systemic Therapy

Patients who do not respond to topical clindamycin are candidates for oral therapy. Tetracyclines are recommended as a first-line treatment for Hurley stage I or II [54]. A common regimen for adults is 100 mg of doxycycline b.i.d. [57]. Except doxycycline, tetracycline (500 mg b.i.d.) or minocycline (100 mg b.i.d.) [58] can be administered. It is contraindicated to take these drugs during pregnancy, and for children under the age of 8 [59]. Common adverse effects include gastrointestinal symptoms and photosensitivity [53]. Erythromycin (500 mg b.i.d.) is also an alternative oral therapeutic option [58]. Oral therapy is typically given for 4 months [53,54].

Clindamycin and rifampicin combination therapy is administered for patients who do not improve with oral tetracyclines treatment, or as first-line treatment for patients in Hurley stage II [53,54]. A typical course includes clindamycin 300 mg b.i.d. and rifampicin 600 mg g.d. (lat. quaque die, once a day) or 300 mg b.i.d. for 10 weeks [53,54]. Before starting this treatment, colitis should be excluded. Common adverse effects include gastrointestinal disorders and orange discoloration of bodily secretions caused by rifampicin [53,54].

Short-term therapy with different antibiotics (i.e., amoxicillin, cephalosporins) can be administered to prevent impairment, or reduce pain [58]. Amoxicillin and clavulanic acid combination has shown to be very effective if administered within the first hour of symptom onset [58]. Combination therapy with rifampicin (300 mg b.i.d./12 weeks), moxifloxacin (400 mg g.d./12 weeks), and metronidazole (400 mg t.i.d./6 weeks) (lat. ter in die, three times a day) has appeared beneficial for more severe cases of HS (Hurley stage II and III) [60]. The main adverse effects of this combination therapy are gastrointestinal disturbances and vulvovaginal candidiasis [53].

Acitretin has been used to treat HS in the early stages of HS (Hurley I or mild II), and the chronic stages of HS with recurrent nodules, abscesses, sinus tracts, and scarring [53]. Acitretin is usually prescribed in doses of 0.25–0.88 mg/kg daily within 3–12 months [53]. Liver enzymes and lipids should be verified 1 month after starting acitretin therapy, and then every 3 months [53]. Pregnancy is contraindicated while taking acitretin, and for 3 years after acitretin cessation [53]. The most common adverse effects of acitretin treatment are retinoid dermatitis symptoms and, among women, hair loss [53]. 

Dapsone is a sulphone drug with antibacterial and anti-inflammatory effects. This drug should be administered if a patient with mild to moderate disease (Hurley stage I or II) fails to respond to the standard first- or second-line treatment [53]. Efficacy (38%) is reported at doses of 25–200 mg daily for at least 3 months. After the improvement, continuous therapy with doses of 50–150 mg daily can be administered to prevent a relapse [58]. Prior to starting dapsone use, baseline hematology, reticulocyte count, liver enzymes, creatinine level, and glucose 6-phosphate dehydrogenase (G6PD) levels should be verified. For follow-ups, assessing hematology, reticulocyte count, liver enzymes, and creatinine levels is required [53].

Zinc salts have anti-inflammatory and antiandrogenic properties, and may be administered as maintenance therapy in Hurley I and II stages [53]. At initiation, 90 mg daily is used. The dose may be lowered according to the efficacy and side effects, mainly gastrointestinal [53].

Ciclosporin A is a calcineurin inhibitor and a potent immunosuppressant. It should be administered to HS patients who fail to respond to standard first-, second-, and third-line therapies [53]. Previously published data reported prescribing ciclosporin A for HS in daily doses of 2–6 mg/kg for a variable duration (6 weeks–7 months) [53]. For follow-ups, assessing blood pressure, hematology, liver enzymes, creatinine levels, and urinalysis is required every other week for the first 3 months, and 3 monthly thereafter [53].

Long-term therapy with systemic corticosteroids can result in rebound flare on withdrawal. On the other hand, short-term systemic therapy (0.5–0.7 mg/kg daily) with rapid tapering can be beneficial in the reduction of lesions in acute flares [53].

### 3.3. Surgical Therapy

Surgical therapy is recommended in more severe cases of HS, with abscesses, fistulas, and sinus tracts formation, or in a longstanding HS refractory to other treatments [58]. The type of surgical procedure and surgical margins are selected based on the stage of HS and body region [54]. For performing surgical procedures, ideally, the disease should be in the remission phase [58].

The surgical treatment of HS includes local procedures, usually performed in the initial disease stages, and extensive procedures, performed in the advanced disease stages [58]. Local surgery includes the incision and drainage of acute lesions, curettage, deroofing, limited local excision of separate lesions, exteriorization or marsupialization, electrocoagulation or radiofrequency sinus ablation, the use of carbon dioxide (CO_2_) laser, long-pulsed neodymium-doped yttrium aluminum garnet (Nd:YAG) laser, and photodynamic therapy (PDT) [53,54,61,62].

Surgical deroofing involves the removal of skin overlying nodules or sinus tracts, and healing by secondary intention. It allows maximal preservation of the healthy tissue between the pathological lesions, and is a good therapy choice for recurrent HS lesions at fixed locations in Hurley stage I or II [53,54].

CO_2_ laser treatment is recommended for HS patients with Hurley stage I or II. This therapy aims at focal radical vaporization of all HS lesions with maximal preservation of the surrounding healthy tissues [53,54].

Extensive interventions in the advanced aggressive disease include excision of smaller areas with primary wound closure; excision of the affected area and closure by secondary healing, without reconstruction; excision of the affected area and reconstruction with immediate or delayed skin grafting (split-thickness skin graft, STSG); and excision of the affected area, and reconstruction with a local, regional, or free flap [53].

Surgical treatments do not provide a 100% safe outcome, and relapses of the disease are possible regardless of the applied method [58].

### 3.4. Adjuvant Therapy

The link between HS, obesity, and cigarette smoking has been well-established by several studies [63]. A general expert opinion is that cessation of cigarette smoking and weight reduction are necessary. Adherence to these guidelines decreases the risk for disease exacerbation [58]. It has been well established that the quality of life is negatively affected by HS. These patients need to be carefully monitored for the possible development of depression and suicidal thoughts [37]. HS lesions are often very painful. It was suggested that ketoprofen patch preparations are beneficial for inflammatory pain treatment, since they have analgesic and anti-inflammatory properties with good skin permeability [53]. Additional exogenous factors contributing to exacerbation of HS are heat, exercise, sweating, stress, fatigue, rubbing, wearing tight clothes, using deodorants, and shaving. Patients should be warned to avoid the above factors as much as possible [54].

## 4. Biologic Therapy

### 4.1. Anti-TNF-α Agents

#### 4.1.1. Adalimumab

Adalimumab is the first fully human recombinant IgG1 monoclonal antibody that binds to soluble and transmembrane TNF-α with high affinity. It is Food and Drug Administration (FDA)-approved for use in rheumatoid arthritis, ankylosing spondylitis, juvenile idiopathic arthritis, psoriatic arthritis, Crohn’s disease, ulcerative colitis, uveitis, and psoriasis [64]. This TNF-α blocker is the only approved biologic drug for the treatment of moderate to severe HS in adults and adolescents after the failure of the conventional treatment [65]. After it received the approval for HS treatment in the USA and Europe from the FDA and European Medical Agency (EMA) in 2015, as well as in Japan in 2019, it became the first approved biologic drug for the treatment of HS worldwide [65]. In October 2018, adalimumab was FDA-approved for HS in ages 12 and up [66].

Two phase III multicenter, double-blind, placebo-controlled studies (PIONEER I and PIONEER II) randomized 633 moderate to severe HS patients to receive adalimumab or placebo. PIONEER I reported that 41.8% of patients treated with adalimumab (160 mg at week 0, 80 mg at week 2, and 40 mg weekly from week 4) achieved HiSCR at week 12, compared to 26.0% in the placebo group. PIONEER II reported that 58.9% of patients treated with the same adalimumab dosage regimen achieved HiSCR at week 12, compared to 27.6% in the placebo group [67]. The improvement in Patient’s Global Assessment of Skin Pain (PGA-SP), mSS, and abscesses and nodule count from the baseline was significant only in the PIONEER II study at week 12 evaluation. However, PIONEER I included patients with more severe disease and higher body mass index (BMI), whereas in the PIONEER II study, patients could continue treatment with tetracyclines [68].

In an open-label extension of PIONEER I and II trials, 88 patients who received 40 mg of adalimumab weekly were followed for a minimum of 60 weeks. At week 36, 62.5% of patients achieved HiSCR, and at week 168, 52.3% of patients maintained HiSCR. The DLQI and mSS improved and maintained through week 72, whereas pain levels improved and remained stable through week 168. The study’s most commonly reported adverse events were nausea and upper respiratory tract infections [69].

A recently published retrospective multicenter cross-sectional study evaluated the biopsychosocial effects of adalimumab treatment in HS patients. Significant improvement in HS-PGA and DLQI scores was reported. Still, patients occasionally reported systemic effects such as depression, development of bowel cancer, and aberrations in liver function tests necessitating drug cessation. Therefore, a holistic approach to follow-up is required [70].

It is well known that patients may exhibit primary nonresponse or secondary loss of response from adalimumab use in HS, as well as in other conditions. Most studies show a positive correlation between adalimumab concentration and optimal clinical outcomes in inflammatory bowel disease (IBD), psoriasis, ankylosing spondylitis, psoriatic, and rheumatoid arthritis [71]. A recently published retrospective case series of 38 HS patients with suboptimal response to adalimumab reported that patients with positive anti-drug antibodies (ADAs), mainly secondary suboptimal responders, have significantly lower serum adalimumab levels [72]. Patients may have subtherapeutic serum drug levels due to high body mass index (BMI), development of ADAs, or high burden of inflammation (elevated CRP). The literature suggests that patients who may benefit from dose escalation are those who have subtherapeutic serum drug levels, and are ADAs-negative. However, patients with supratherapeutic serum drug levels are not candidates for further dose escalation because of the increased risk for systemic side effects with minimal clinical benefit [72]. Therefore, therapeutic drug monitoring (TDM) could be suggested as an option in detecting HS patients who may benefit from dose escalation, thus limiting side effects that may occur due to empiric dose escalation.

In a multicenter cohort study, an inverse correlation between the adalimumab delay and significant clinical response was observed. Proof for a ‘window of opportunity’, when it comes to therapy of HS, supports early adalimumab use [73].

Adalimumab is usually well-tolerated among patients. Relatively common adverse side effects are injection site reactions. Rare, but serious, adverse effects of adalimumab include pneumonia, septic arthritis, diverticulitis, pyelonephritis, tuberculosis, or hepatitis B reactivation, as well as neurological and hematological complications [74]. Very rarely, malignancies (including lymphomas and non-melanoma skin cancers) can occur [75]. A few cases of paradoxical development of HS during the treatment with adalimumab, administered due to other diseases, were reported [76]. A study that evaluated the safety data of adalimumab concluded that the safety of adalimumab administered every week and every other week was comparable and consistent with the expected adalimumab safety profile [77].

A retrospective observational study evaluated the efficacy and safety of adalimumab biosimilar (SB5) in patients with moderate to severe HS. It included four adalimumab naive patients and seven patients who were switched from the adalimumab originator. Results showed that adalimumab SB5 is as effective and well-tolerated as its originator for the treatment of HS (Table 3) [78].

#### 4.1.2. Infliximab

Infliximab is a chimeric mouse/human IgG1 monoclonal that binds to soluble and transmembrane TNF-α with high affinity. It is FDA-approved for use in IBD, rheumatoid arthritis, ankylosing spondylitis, psoriatic arthritis, and plaque psoriasis [79].

In a phase II double-blind, placebo-controlled trial, 38 patients with moderate to severe HS were randomized to receive 5 mg/kg of intravenous infliximab on weeks 0, 2, 4, 6, 14, and 22, or a placebo. By week 8, 57% of patients had a 50% or greater decrease in HSSI compared to 5% of patients in the placebo group. The infliximab use also improved the pain VAS and DLQI scores [80].

A long-term study evaluated the efficacy of a single course of infliximab (5 mg/kg intravenously at weeks 0, 2, and 6) on 10 patients, and followed up for at least 1 year. Three patients had no recurrence of lesions in a 2-year follow-up period [81].

A study conducted to determine the optimal dosing of infliximab included 52 patients with HS. Patients tolerated infliximab well, and achieved significant improvement in abscess and nodule count, most of them (67%) at a 10 mg/kg schedule intravenously every 6 or 8 weeks [82]. A prospective analysis of 42 HS patients treated with infliximab concluded that administration of infliximab 7.5 mg/kg intravenously every 4 weeks, with the dose increasing to 10 mg/kg intravenously if necessary, provides optimal reduction of HS-related disease activity [83].

Several case reports described the occurrence of demyelinating neuropathies, *Gemella morbillorum* bacteremia, and a metastatic cutaneous squamous cell carcinoma during infliximab treatment [84,85,86].

A retrospective multicenter study assessed the survival of adalimumab and infliximab in a daily practice of HS patients, and identified predictors for drug survival. Survival rates, mainly determined by ineffectiveness, were similar for adalimumab and infliximab after 12 months. Age, disease duration, and surgery are recognized as predictors for longer survival time [87].

In a retrospective cohort study of 34 HS patients, the infliximab efficacy was compared to its biosimilar infliximab-abda. Both drugs showed equally good clinical results (Table 3) [88].

#### 4.1.3. Etanercept

Etanercept is a fully human recombinant molecule consisting of two soluble TNF receptor subunits (p75) fused to the Fc portion of human IgG1. It is FDA-approved for use in rheumatoid arthritis, juvenile idiopathic arthritis, ankylosing spondylitis, plaque psoriasis, and psoriatic arthritis **[89]**.

In a phase II placebo-controlled study, 20 patients with moderate to severe HS were randomized to receive etanercept 50 mg or placebo subcutaneously twice a week for 12 weeks. Afterwards, 50 mg of etanercept subcutaneously twice a week for 12 more weeks was administered to all participants. The results showed that etanercept was not efficient enough for HS treatment (Table 3) [90].

#### 4.1.4. Golimumab

Golimumab is a fully human anti-TNF-α IgG1 monoclonal antibody that binds to soluble and membrane-bound TNF-α with high affinity. It is FDA-approved for use in rheumatoid arthritis, psoriatic arthritis, ankylosing spondylitis, and ulcerative colitis [91]. 

Our literature search found only two case reports regarding the use of golimumab for HS. One described the use of golimumab, 50 mg subcutaneously every 4 weeks, in a patient with severe HS and psoriatic arthritis who had failed adalimumab and anakinra previously. After administering golimumab, the patient’s HS worsened despite the improvement in her psoriatic arthritis [92]. The second case report described a patient with HS, pyostomatitis vegetans, and ulcerative colitis who experienced remission of HS within 2 months of starting golimumab treatment (200 mg at week 0, and then 100 mg every 4 weeks) (Table 3) [93].

#### 4.1.5. Certolizumab Pegol

Certolizumab pegol (CZP) is a pegylated humanized monoclonal anti-TNF-α agent, which lacks the fragment crystallizable (Fc) region, thus preventing active placental transfer. It is FDA-approved for use in Crohn’s disease, rheumatoid arthritis, psoriatic arthritis, ankylosing spondylitis, and plaque psoriasis [94].

Several case reports described the use of certolizumab pegol (400 mg subcutaneously every other week) in HS patients refractory to other biological drugs with good disease control [95,96,97].

A retrospective study on the use of TNF-α inhibitors described no efficacy of certolizumab pegol (200 mg subcutaneously every 2 weeks) treatment in two HS patients [98].

One of the published case reports described the treatment of a pregnant HS patient with certolizumab pegol. From 19 weeks of gestation, certolizumab pegol 400 mg was administered subcutaneously every other week. At the 8-week follow-up, the patient achieved HiSCR. However, at the next visit, the patient presented with worsening pain and one new abscess. Therefore, the dose was increased to 400 mg subcutaneously weekly at 32 weeks of gestation, and symptoms significantly improved. The medication was well-tolerated, and adequate pain control was achieved (Table 3) [95].

### 4.2. Anti-IL-17 Agents

#### 4.2.1. Secukinumab

Secukinumab is a fully human IgG1κ monoclonal antibody that binds to IL-17A with high affinity. It is FDA-approved for moderate to severe plaque psoriasis, psoriatic arthritis, and ankylosing spondylitis [99].

In a 24-week-long, open-label pilot trial of nine patients with moderate to severe HS, secukinumab 300 mg was administered subcutaneously weekly for 5 weeks, and then every 4 weeks for 24 weeks. Even though HiSCR was achieved in 78% of patients by week 24, the authors concluded that the psoriasis dosing regimen might not be sufficient for treating HS, as inflammatory levels are higher in HS [100].

In another 24-week-long, open-label trial of 20 patients with moderate to severe HS, secukinumab at two dose levels was administered. After an induction dose of secukinumab 300 mg subcutaneously weekly for 5 weeks, nine patients were administered secukinumab 300 mg subcutaneously every 4 weeks, and eleven patients were administered secukinumab 300 mg subcutaneously every 2 weeks. Seventy percent (*n* = 14) of all patients achieved HiSCR by week 24 [101]. These results indicate that increased frequency of secukinumab administration does not result in better clinical outcomes, and rather could increase the risk of possible side effects.

In a retrospective cohort study, which included 20 HS participants, secukinumab 300 mg was administered subcutaneously weekly for 5 weeks, and then every 4 weeks up to 16 weeks. Seventy-five percent of patients achieved a successful HiSCR response by week 16. No relapse was observed during an average follow-up of 14 months after secukinumab treatment. However, two patients with no personal or family history of IBD developed Crohn’s disease (CD) after 3 and 5 months of treatment [102]. Due to the reported onset of paradoxical events (IBD exacerbation) after the treatment of IBD with anti-IL-17 agents [103], caution is necessary for HS patients with an increased risk for IBD when initiating an anti-IL-17 treatment [102]. The results of a large cross-sectional study of 3207 HS patients in Israel demonstrated a significant association between HS and CD, but not between HS and ulcerative colitis [104].

An Italian retrospective study evaluated the efficacy of secukinumab (300 mg every week at weeks 0, 1, 2, 3, 4, and then every 4 weeks) in HS patients. It was reported that 41% of patients achieved HiSCR at week 28 [105].

More paradoxical events regarding the use of secukinumab were described, including a case of secukinumab-induced HS in a psoriasis patient, and a case of a psoriasiform eruption caused by adalimumab in an HS patient, which was controlled afterwards by secukinumab [106].

Three phase III randomized placebo-controlled trials to assess efficacy and safety of two secukinumab dose regimens in HS patients are currently underway (NCT03713619, NCT03713632, NCT04179175) (Table 4) [107,108,109].

#### 4.2.2. Brodalumab

Brodalumab is a fully human IgG2 monoclonal antibody that targets the A subunit of the IL-17 receptor, thus blocking the signaling of multiple isoforms of IL-17. It is FDA-approved for moderate to severe plaque psoriasis in people who have not improved with other treatments [110].

An open-label cohort study of 10 patients with moderate to severe HS with no previous history of IBD evaluated the efficacy of brodalumab (210 mg subcutaneously at weeks 0, 1, and 2, and then every 2 weeks). All patients achieved HiSCR in week 2. However, deeper nodules and abscesses resolved after more than 12 weeks, and two participants with severe HS experienced higher pain levels. Also, an increase in draining tunnel counts at week 4 was observed, which then continued to decrease over time [111].

In another open-label cohort study of 10 HS patients, the authors hypothesized that every-week brodalumab dosing might provide better disease control compared to every-other-week dosing. Brodalumab 210 mg was administered subcutaneously weekly for 24 weeks. All patients achieved HiSCR at week 4, 80% achieved a 75% reduction in total abscesses and nodules count (HiSCR 75), and 50% achieved a 100% reduction in total abscesses and nodules count (HiSCR 100) at week 12. By week 24, all patients maintained HiSCR. There was no recurrence in draining tunnels [112].

To better characterize the response to weekly treatment of brodalumb, the recruitment of patients into a new phase I clinical trial is ongoing (NCT04979520) (Table 4) [113].

The extremely rapid clinical response of 20 HS patients treated with brodalumab and a significant reduction in dermal tunnel drainage [111,112] are worthy of attention. These results might occur due to the blockade of three IL-17 isoforms (IL-17A, IL-17C, and IL-17F) by brodalumab [110]. However, larger randomized placebo-controlled trials are required to investigate whether brodalumab is indeed such an effective treatment for HS.

#### 4.2.3. Bimekizumab

Bimekizumab is a humanized IgG1κ monoclonal antibody that neutralizes both IL-17A and IL-17F. Studies have shown that bimekizumab is effective in patients with psoriasis [114].

A phase II clinical trial randomized 90 patients with moderate to severe HS to receive bimekizumab (640 mg at week 0, and then 320 mg every 2 weeks), a placebo, or adalimumab. At week 12, 46% of patients treated with bimekizumab achieved a 75% reduction in total abscesses and nodules count (HiSCR 75), and 32% of them achieved HiSCR 90, in comparison to 10% of patients treated with placebo who achieved HiSCR 75, and none of whom achieved HiSCR 90. Thirty-five percent of patients treated with adalimumab achieved HiSCR 75, and 15% of them achieved HiSCR 90 [115].

Currently, three phase III clinical studies to evaluate the efficacy and safety of bimekizumab in HS treatment are underway (NCT04242446, NCT04242498, NCT04901195) (Table 4) [116,117,118].

#### 4.2.4. Ixekizumab

Ixekizumab is a humanized IgG4 monoclonal antibody that neutralizes soluble IL-17A and IL-17 A/F. It is FDA-approved for use in moderate to severe plaque psoriasis, active psoriatic arthritis, active ankylosing spondylitis, and active non-radiographic axial spondyloarthritis with objective signs of inflammation [119].

Two case reports of HS patients with concomitant psoriasis treated with ixekizumab were published. The results suggested ixekizumab as a safe and effective treatment for both psoriasis and HS [120,121].

Another case report of a female HS patient with concomitant herpes simplex virus (HSV) infection reported ixekizumab administration. After HSV resolution, the patient was initiated on subcutaneous ixekizumab 160 mg at week 0, and 80 mg at weeks 2, 4, 6, 8, 10, and 12, followed by 80 mg every 4 weeks. Thirteen months after initiating ixekizumab, the patient demonstrated significant improvement in her condition, suggesting that ixekizumab may represent another promising therapeutic modality (Table 4) [122].

#### 4.2.5. CJM112

CJM112 is a human IgG1κ monoclonal antibody that neutralizes soluble IL-17 and IL-17A/F [123].

A phase II randomized controlled study was conducted in patients with moderate to severe HS to evaluate the efficacy and safety of multiple doses of CJM112 (NCT02421172). HS-PGA response rate was 32.3% in patients treated with CJM112 at week 16, compared to 12.5% in patients treated with placebo (Table 4) [124].

### 4.3. Anti-IL-12/23 Agents

#### Ustekinumab

Ustekinumab is a fully human IgG1κ monoclonal antibody that neutralizes the p40 subunit of IL-12 and IL-23. It is FDA-approved for use in plaque psoriasis, psoriatic arthritis, and Crohn’s disease [125]. 

A phase II open-label study included 17 patients with moderate to severe HS treated with 45 or 90 mg ustekinumab subcutaneously at weeks 0, 4, 16, and 28. In 82% of patients, moderate to marked improvement of the mSS was reported at week 40, whereas 47% of patients achieved HiSCR. A low concentration of leukotriene A4-hydrolase (LTA4H) was observed in good responders to the biologic, which may be predictive of ustekinumab effectiveness [126].

A multicentric retrospective review reported treatment of 14 HS patients with an ustekinumab dosage regimen for Crohn’s disease. Patients received an intravenous infusion of ustekinumab adjusted by weight (≤55 kg, 260 mg; 55–85 kg, 390 mg; ≥85 kg, 520 mg), and then a subcutaneous maintenance dose of 90 mg every 8 weeks. The study involved the patients with recalcitrant HS, resistant to at least one prior biologic drug. Fifty percent of the patients achieved HiSCR. A significant pain reduction and quality of life improvement were observed in 71.42% of patients at week 16 [127]. The equal dosing schedule was administered in a prospective study of six HS patients with similar results (50% of patients achieved HiSCR at week 12) [128].

Several case reports and case series studies described the variable efficacy of ustekinumab in treating HS patients. In one case series study, the improvement in the HS-PGA score was noticed in 70% of patients, and the improvement in the Numerical Pain Rating Scale (NPRS) was recorded in 80% of patients [129]. Another case series study reported that 9 out of 10 patients treated with ustekinumab 90 mg subcutaneously every 8 weeks without an induction dose reached the HiSCR with an improvement in Hurley staging system and HS-PGA score, together with a decrease in monitored analytical parameters (Table 5) [130].

Based on the published results so far, it cannot be concluded that the ustekinumab dosage regimen for Crohn’s disease results in better clinical outcomes than the psoriasis dosage regimen. However, this requires further investigation in randomized controlled trials.

### 4.4. Anti-IL-23 Agents

#### 4.4.1. Guselkumab

Guselkumab is a human IgG1κ monoclonal antibody that neutralizes the p19 subunit of IL-23. It is FDA-approved for use in adults with moderate to severe plaque psoriasis [131].

Several case reports and case series reported achieving HiSCR after administering 100 mg guselkumab subcutaneously at weeks 0 and 4 (induction phase), followed by injections every 8 weeks in HS patients [132,133,134,135,136].

In a case series that included four patients, subcutaneous injections of 100 mg guselkumab were administered in higher frequency at week 0, and then every 4 weeks. Two patients (50%) only slightly improved after the guselkumab treatment, one patient showed no response to therapy, and one patient had increased HS severity after the treatment [137].

In a phase II placebo-controlled, double-blind study (NCT03628924), patients with moderate to severe HS were randomized to receive two different dosages of guselkumab or a placebo. The results showed that HiSCR was achieved at week 16 in 50.8% of the participants treated with 200 mg of guselkumab subcutaneously at weeks 0, 4, 8, and 12. In comparison, 38.7% of the participants who received a placebo, and 45% of participants who received 1200 mg of guselkumab intravenously at weeks 0, 4, and 8, followed by 200 mg of guselkumab subcutaneously at week 12, achieved HiSCR at week 16 (Table 6) [138].

#### 4.4.2. Risankizumab

Risankizumab is a fully human IgG1κ monoclonal antibody that neutralizes the IL-23 [139]. It is FDA-approved for use in adults with psoriasis and/or psoriatic arthritis [140].

We found four case reports describing the successful use of risankizumab (150 mg administered subcutaneously at weeks 0, 4, and then every 12 weeks) in HS patients [141,142,143].

In a phase II placebo-controlled study, patients with moderate to severe HS were randomized to receive one of two dose levels of risankizumab or a placebo. No results have been posted yet (NCT03926169) (Table 6) [144].

#### 4.4.3. Tildrakizumab

Tildrakizumab is a humanized IgG1κ monoclonal antibody that neutralizes the p19 subunit of IL-23. It has been FDA-approved for use in moderate to severe plaque psoriasis [145].

As found by our literature search, there are only two case series studies describing the use of tildrakizumab 100 mg subcutaneously at weeks 0 and 4, and then 200 mg every 4 weeks, for the treatment of moderate to severe HS. All patients achieved HiSCR at week 8, and half of them from every study reported improvement in DLQI scores (Table 6) [146,147].

### 4.5. Anti- IL-1 Agents

#### 4.5.1. Anakinra

Anakinra is a fully human recombinant IL-1 receptor (IL-1R) monoclonal antibody that inhibits IL-1α and IL-1β from interacting with IL-1R. It is FDA-approved for use in rheumatoid arthritis, neonatal-onset multisystem inflammatory disease, and deficiency of IL-1 receptor antagonist (DIRA) [148,149].

In a 24-week-long, placebo-controlled trial, 20 HS patients were randomized to receive 100 mg of anakinra subcutaneously g.d. or a placebo for 12 weeks. At week 12, 78% of HS patients treated with anakinra achieved HiSCR, compared to 30% in the placebo group. However, the difference in the HiSCR rates after 12 weeks was not significant [150].

A case series of six HS patients treated with 100 mg of anakinra subcutaneously g.d. showed improvement in the mSS, HS-PGA, and DLQI scores after 8 weeks for five patients who completed the treatment course. However, relapse of HS occurred eight weeks after the end of the treatment course [151].

Due to the reported rapid rebound of HS after anakinra cessation [150,151], long-term safety and maintenance of clinical improvement of this drug in HS patients should be investigated in further randomized controlled trials.

One case report described the administration of higher doses of anakinra (200 mg subcutaneously g.d.) to a female patient with refractory HS. This led to the disease remission, which was maintained 1 year after the end of anakinra treatment [152].

Several case reports reported the failure of anakinra in treating severe HS (Table 7) [92,153,154].

#### 4.5.2. Bermekimab

Bermekimab, also named MABp1, is a fully human recombinant IgG1κ monoclonal antibody that neutralizes IL-1α [155]. High concentrations of IL-1β and IL-1α in HS lesional skin suggest that IL-1α is also involved in the inflammatory process of HS [156].

In a prospective trial, 20 HS patients were randomized to receive a placebo or bermekimab (7.5 mg/kg intravenously every other week) for 12 weeks. By the end of week 12, 60% of patients treated with bermekimab achieved HiSCR, compared to 10% of patients treated with placebo (NCT02643654) [156]. When results of the trial became available, patients initially randomized to placebo treatment were allowed to continue in an open-label extension. Eight of them received bermekimab 7.5 mg/kg intravenously every other week for 12 weeks. Fulfillment of HiSCR was achieved in 75% of patients at the end of the study [157].

In a recent phase II open-label study, HS patients who were naive to anti-TNF therapy (18), or had failed prior anti-TNF therapy (23), were treated with 400 mg of bermekimab subcutaneously weekly (13 doses). A high percentage of patients achieved HiSCR at week 12 (61% of patients who were naive to anti-TNF therapy, and 63% of those who had failed anti-TNF therapy) [158].

A new, phase II, randomized, placebo and active comparator-controlled dose range study to evaluate the safety and efficacy of bermekimab for the treatment of HS patients is currently recruiting participants (NCT04988308) (Table 7) [159].

Further phase II and phase III randomized controlled studies are necessary to confirm the efficacy and safety of this promising biologic in the HS treatment.

#### 4.5.3. Canakinumab

Canakinumab is a fully human IgG1κ monoclonal antibody that targets IL-1β. It is FDA-approved for use in cryopyrin-associated periodic syndromes and systemic juvenile idiopathic arthritis [160].

Our literature search found controversial results regarding canakinumab use (150 mg on day 0, and monthly afterwards) in HS patients. Several case reports described a positive response, demonstrated by an improvement in mSS and reduced pain score without any adverse effect [161,162], whereas others reported a negative response [163,164] (Table 7).

## 5. Other Immunomodulatory Therapy

### 5.1. Phosphodiesterase-4 (PDE-4) Inhibitor

#### Apremilast

Apremilast is an orally administered selective PDE-4 inhibitor that regulates inflammatory mediators, such as TNF-α, IL-10, and IL-23. It is FDA-approved for use in patients with moderate to severe plaque psoriasis and active psoriatic arthritis [13].

A trial in which 20 patients with moderate to severe HS were randomized to receive either 30 mg of apremilast b.i.d. (15 patients) or placebo (5 patients) reported that 53.3% of patients treated with apremilast achieved HiSCR at week 16 compared to 0% of patients treated with placebo [165].

After the study completion, 100% of the responders chose to continue treatment. Fifty percent (4/8) of initial responders discontinued treatment within the first year, and the 2-year follow-up data were available for four of the initial responders. All patients who continued the treatment maintained HiSCR during the 2-year follow-up [166].

In a case series study of nine HS patients who failed to respond to other treatments, five out of six patients who persisted with treatment showed a significant improvement in the mSS, the pain VAS, and DLQI scores [167].

### 5.2. Complement C5a Inhibitors

#### 5.2.1. Vilobelimab (IFX-1)

IFX-1 is an IgG4κ monoclonal antibody that binds to C5a with high affinity, and blocks its biological activity [168].

An open-label trial, which included 12 HS patients, reported that 75% of patients treated with IFX-1 achieved HiSCR after 50 days. After 134 days, this increased to 83.3% [168].

A phase II controlled trial (NCT03487276) randomized the patients with moderate to severe HS to receive four different doses of IFX-1 intravenously (400 mg every 4 weeks, 800 mg every 4 weeks, 800 g every 2 weeks, and 1200 mg every 2 weeks) or placebo. The highest HiSCR response rate (51.5%) was achieved after vilobelimab 800 mg intravenously every 4 weeks. The HiSCR response rate in patients treated with placebo was 47.1% [169].

#### 5.2.2. Avacopan

Avacopan is an orally administered small molecule C5a receptor antagonist. Several studies are underway for the treatment of HS, antineutrophil cytoplasmatic antibodies-associated vasculitis, and atypical hemolytic uremic syndrome with avacopan [170].

A placebo-controlled trial (NCTO3852472) randomized HS patients into three groups (placebo, avacopan 10 mg orally b.i.d., and avacopan 30 mg orally b.i.d.). The results of this study are yet to be published [171].

### 5.3. Inhibitors of Janus Kinase (JAK) Family

#### 5.3.1. Janus Kinase (JAK) Inhibitors

Signals from IL-2 receptor (IL-2R), IL-4 receptor (IL-4R), IL-5 receptor (IL-5R), IL-6 receptor (IL-6R), IL-13 receptor (IL-13R), and type I interferons activate JAKs, which subsequently activate signal transducers and activators of transcription proteins (STATS). After activation, STATs enter the nucleus to bind to transcriptional regulatory sites of target genes, and induce inflammation. Several studies evaluated the efficacy of JAK inhibitors in HS [172].

INCB054707 is a JAK1 inhibitor assessed in two phase II trials (NCT03569371 [173] and NCT03607487 [174]) in patients with moderate to severe HS. In the NCT03607487 study, patients were randomized to receive INCB054707 orally g.d. in three different doses (30, 60, 90 mg) or placebo. Eighty percent of patients treated with 30 and 60 mg of INCB054707 had grade 1–2 adverse events (adverse events that do not result in death, are not life-threatening, and do not require inpatient hospitalization). Fifty percent of patients treated with 90 mg had grade 1–2 adverse events, and 37% of patients had grade 3 adverse events (adverse events that result in death, are life-threatening, or require inpatient hospitalization) [174]. Another phase II trial (NCT04476043) is currently underway to evaluate the efficacy and safety of INCB054707 in HS patients [175].

A phase II randomized placebo-controlled trial (NCT04430855) evaluated how well upadacitinib works to treat adult patients with moderate to severe HS. The results of this study are yet to be published [176].

Tofacitinib is a JAK inhibitor that has been used with good clinical outcomes in two HS patients resistant to other biologics [177].

#### 5.3.2. IL-1 Receptor-Associated Kinase 4 (IRAK4) Inhibitors

IRAK4 functions downstream of multiple innate immune cell receptors, such as TLR and IL-1Rs [172].

KT-474 is an orally administered small molecule that targets IRAK4, and is under development for the treatment of interleukin-1 receptor (IL-1R)/ TLR-driven inflammatory diseases [172]. A phase I study (NCT04772885) is currently underway, and is going to assess the safety, tolerability, pharmacokinetics, and pharmacodynamics of KT-474 in patients with HS or atopic dermatitis (AD) [178].

PF-06650833 is an IRAK4 inhibitor [172] investigated in a phase II study (NCT04092452) among participants with moderate to severe HS, which assessed the safety and efficacy of three kinase inhibitors (PF-06650833, PF-06700841, and PF-06826647) [179].

#### 5.3.3. Tyrosine Kinase 2 (TYK2) Inhibitors

Signals from cytokines IL-23, IL-12, and type I interferons activate TYK2 [172]. Ropsacitinib (PF-06826647) is a tyrosine kinase 2 (TYK2) inhibitor [172] and a part of the above-mentioned phase II study with three kinase inhibitors (NCT04092452) [179]. Except for HS, it is being investigated for psoriasis and ulcerative colitis [39].

#### 5.3.4. Tyrosine Kinase 2 (TYK2) Inhibitors/Janus Kinase 1 (JAK1) Inhibitors

Brepocitinib (PF-06700841) is a TYK2/JAK1 inhibitor that prevents IL-12 and IL-23 signaling [172]. Several studies are conducted to assess the use of brepocitinib for the treatment of HS and psoriasis [39]. It is a part of the above-mentioned phase II study with three kinase inhibitors (NCT04092452) [179].

### 5.4. CD-20 Inhibitor

#### Rituximab

Rituximab is a chimeric mouse/human monoclonal antibody that targets CD20 protein. It is FDA-approved for use in non-Hodgkin’s lymphoma, chronic lymphocytic leukemia, rheumatoid arthritis, granulomatosis with polyangiitis, microscopic polyangiitis, and pemphigus vulgaris [180].

A recent case report described rituximab administration in low doses (200 mg intravenously in two courses) in a kidney transplant recipient with idiopathic carpotarsal osteolysis who suffered from chronic active antibody-mediated rejection (CAAMR). After the kidney transplantation, the patient was diagnosed with HS. Dramatic improvement of HS was reported after the rituximab administration, but the dose was insufficient for CAAMR [181].

### 5.5. CD-40 Inhibitor

#### Iscalimab (CFZ533)

CFZ533 (iscalimab) is a fully human monoclonal antibody that blocks the CD40 pathway [172]. A phase II clinical study (NCT03827798) to evaluate the efficacy and safety of CFZ533 in HS patients is currently being conducted [182].

### 5.6. Anti IL-36 Agents

#### 5.6.1. Spesolimab

Spesolimab is a humanized monoclonal antibody that blocks the activation of the IL-36 receptor (IL-36R) [172], and is presently being investigated in phase II trials for moderate to severe palmoplantar pustulosis [183]. A randomized phase II trial (NCT04762277) is currently underway to evaluate the efficacy of spesolimab in HS patients [184].

#### 5.6.2. Imsidolimab (ANB019)

Imsidolimab is a humanized monoclonal antibody that blocks the activation of IL-36R [39]. It is currently being evaluated in a phase II trial (NCT04856930) [185].

### 5.7. Leukotriene A4 (LTA4) Inhibitor

#### LYS 006

LYS 006 is an orally administered LTA4 hydrolase inhibitor that is currently being investigated for the treatment of HS and inflammatory acne [172]. A phase II clinical study (NCT03827798) is underway to evaluate the efficacy and safety of LYS 006 in HS patients [182].

### 5.8. Inhibitor of Chemokines That Bind to CXCR1 and CXCR2 Receptors

#### LY 3041658

LY 3041658 is a monoclonal antibody that neutralizes chemokines that bind to the CXCR1 or CXCR2 receptors [39]. A phase II randomized controlled study is currently underway to evaluate the efficacy of LY 3041658 in patients with moderate to severe HS (NCT04493502) [186].

### 5.9. Anti-Granulocyte Colony-Stimulating Factor (G-CSF) Agent

#### CSL 324

CSL 324 is a recombinant anti-G-CSF receptor monoclonal antibody that might be effective in treating diseases associated with increased numbers of neutrophils at sites of inflammation [68]. A phase I study (NCT03972280) is currently underway to evaluate the pharmacokinetics and safety of repeating doses of CSL 324 in participants with HS and palmoplantar pustulosis [187].

## 6. Discussion

Currently, there are several available treatment options for HS. A combination of conservative therapy and surgical treatment ensured the best clinical outcome before the biologic era. Nowadays, biologic therapy is the most effective pharmacological treatment of moderate to severe HS [53].

An anti-TNF-α biologic, adalimumab, was approved by the FDA and the EMA 7 years ago, and has significantly improved the clinical outcomes in HS patients since then [65]. However, the long-term effectiveness of this TNF-α inhibitor in HS patients has shown to be highly variable. Due to a significant need to improve the quality of life of HS patients, a search for new targeted therapy increased. This article presents current and future therapeutic targets, with a special accent on biologics as the most promising therapeutic options.

Most exposure–response relationship studies show a positive correlation between biologics concentration and optimal clinical outcomes in immune-mediated diseases [71]. One of the most significant adverse events of biologics is the formation of ADAs, which results in decreased drug concentration, and a suboptimal clinical response [188]. Across immune-mediated diseases, including psoriasis, IBD, rheumatoid arthritis, psoriatic arthritis, juvenile idiopathic arthritis, ankylosing spondylitis, and non-radiographic axial spondyloarthritis, infliximab, a chimeric mouse/human antibody, was associated with the highest, and secukinumab with the lowest rates of ADAs [189]. A recently published systematic review compared the frequency of ADAs development in HS patients treated with adalimumab or infliximab. The immunogenicity of infliximab was significantly higher than that of adalimumab (frequency of ADAs was 47–60% in the infliximab group vs. 7.4–10.7% in the adalimumab group). However, a significant bias of this comparison included different drug regimens, differences in disease severity, and different methods of measuring ADAs [188]. The literature suggests that the prevalence of ADAs can vary depending on the used assay. In most assays, ADAs detection is compromised due to the formation of immune complexes between adalimumab and ADAs [190]. Further studies are necessary to develop an antibody assay applicable for clinical testing, which overcomes the limitation of therapeutic response.

Combination therapy with a TNF-α inhibitor and an oral immunomodulator is often utilized for patients with rheumatoid arthritis or IBD to enhance the efficacy of the biologic drug, or reduce the risk of immunogenicity [72]. Methotrexate has been found to induce anergy, where T and B cells fail to react to an antigen, as shown in mouse models [188]. Due to the reported possible formation of ADAs in secondary suboptimal responders to adalimumab with HS [72], further studies are necessary to determine whether co-treatment with an oral immunomodulator (i.e., methotrexate) reduces the risk of immunogenicity, as well as whether the dose escalation is successful in HS patients with subtherapeutic adalimumab levels and no detectable ADAs. TDM could be a reasonable option for extending dosing and limiting side effects on a per-patient level.

Since HS has some similar features to psoriasis, the identification of potential drugs for HS has often been based on preexistent drugs for psoriasis rather than on HS pathogenesis [68]. Although the existence of common pathogenetic pathways in HS and psoriasis has been proven on multiple occasions, the fact remains that the same biologic therapy is shown to be less effective when treating HS patients than psoriasis patients [7].

The registration of a new biologic drug requires evidence for effectiveness and safety in randomized controlled trials [191]. However, the inclusion criteria for randomized clinical studies have mostly included patients with scarring components (which excludes mild disease), and the exclusion criteria were often applied to patients who need the most demanding therapy (Hurley III) [191]. Therefore, this can cover up the true effectiveness of the drug.

Most biologics and other investigated immunomodulatory therapies for HS have a generally good safety profile [192]. However, safety concerns, including infection risks, the development of malignancy, and demyelinating disorders, are still waiting to be evaluated, especially in the light of more intensive dosing regimens of biologics in HS than in psoriasis [192].

Presently, the most promising biologics in phase III trials are anti-IL-17, secukinumab, and bimekizumab. The biologic currently in phase II trials that targets IL-1, bermekimab, shows encouraging results too. Treatment should be started as early as possible to prevent fibrosis and sinus tracts formation. An inverse correlation was established between adalimumab use and clinical response [73].

HS patients commonly have other immune-mediated diseases, so determining the optimal therapy for both HS and comorbidities is often a real-life problem. A round-table session held in 2020 during the 9th Conference of the European Hidradenitis Suppurativa Foundation in Athens concluded that targeting TNF-α and IL-12/23 results in better clinical outcomes in psoriasis than in psoriatic arthritis, HS, and Crohn’s disease, whereas targeting IL-17 is significantly more effective in psoriasis than in psoriatic arthritis, and it is obviously not effective in Crohn’s disease [193]. Further head-to-head clinical trials are necessary due to variable clinical responses to biologics determined by the drug mechanism and by the disease being treated.

The additional complications that may occur during the treatment with biologic drugs are paradoxical responses, i.e., when targeting an involved cytokine may exacerbate another disease originally mediated through the same cytokine. For example, according to our experience, paradoxical development of psoriasis may develop in patients treated with adalimumab due to their HS; and *vice versa*, patients with psoriasis may develop HS during adalimumab treatment. Other authors reported similar experiences with adalimumab [76]. In addition, there are reports of secukinumab-induced HS in a psoriasis patient [106]. Several hypotheses were proposed to explain the onset of paradoxical HS during the treatment with adalimumab administered for other conditions. The possible pathogenetic factors include modification of cytokine balance, activation of alternate pathways such as IL-1β, and potential occult infection, a well-known trigger for HS [194]. Therefore, caution is necessary when administering biological drugs, particularly the new ones, to detect the occurrence of new or as yet undescribed paradoxical events.

Biologics are often used before the surgery to minimize the area required for surgical resection in patients with severe HS that do not respond well enough to biologics alone [13]. The results from the phase IV trial (NCT02808975) that assessed the efficacy and safety of adalimumab and surgery combination could help to improve the treatment approach for severe HS [195].

Successful use of the new therapeutic options depends on the proper stratification of patient groups. Clinically relevant biomarkers could be helpful. A recent systematic review critically evaluated biomarkers in HS, and showed that only one diagnostic (serum IL-2R), one monitoring (dermal Doppler vascularity), and two predictive biomarkers (epithelialized tunnels and positive family history of HS) achieved a high GRADE (grading of Recommendations, Assessment, Development, and Evaluation), but none of them had sufficient clinical validity to be recommended for routine use in the clinical setting [196].

The abundance of different methods measuring clinical outcomes of new, targeted treatments for the HS has led to difficulties in comparing the efficacy of different drugs. HiSCR, the most commonly used validating score in randomized controlled trials, is more dynamic than Hurley staging, easy to perform contrary to mSS, and in good correlation with patient-reported outcomes. The main disadvantage of HiSCR is in high placebo rates in randomized controlled trials, probably due to its construction as a binary variable [193]. An ideal severity scoring system needs to be validated against other physician- and patient-reported outcomes, easy to perform both in clinical trials and daily practice, dynamic with a clear deterioration between mild and moderate to severe disease, and consensus-based [193]. IHS4 considers nodules, abscesses, and draining fistulas, with the latter as a negative predictor for mild HS. However, draining fistulas are not necessary to classify patients with the moderate disease [11]. On the contrary, the Hurley II stage requires the presence of draining fistulas/sinus tracts [9], suggesting that IHS4 can be a more representative outcome measure than both HiSCR and Hurley staging systems. Therefore, new studies on the use of IHS4 in clinical trials are necessary.

Further research on novel therapeutic agents using validated HS scoring systems, further longitudinal studies assessing efficacy and safety of new drugs, and proper stratification of patient groups, hopefully with the help of HS biomarkers, are required to achieve adequate clinical response, and to improve the quality of life of HS patients significantly.

## 7. Materials and Methods

The literature search was conducted using the PubMed and Google Scholar repositories. Only relevant meta-analyses, observational studies, randomized controlled trials, and systematic reviews published in English and Croatian and between 1998–2022 were taken into consideration, utilizing the keywords: hidradenitis suppurativa, pathophysiology, treatment, biologics, TNF-α inhibitors, IL-17 inhibitors, IL-12/23 inhibitors, IL-23 inhibitors, IL-1 inhibitors, JAK inhibitors, small molecule inhibitors. An additional review of phase I, II, and III randomized clinical trials registered at Clinicaltrials.gov was conducted up to February 2022, and all of the studies evaluating the efficacy and safety of biologic drugs and small molecule therapeutics were included in the review. Two authors (A.M.Č. and Z.B.M.) collected sources of information, read the reference abstracts, and included a total of 196 articles and published results of registered randomized controlled trials primarily focusing on the molecular pathophysiology of hidradenitis suppurativa, conservative therapy of hidradenitis suppurativa, as well as current biologic and other immunomodulatory therapeutics used to target underlying pathogenesis of HS. After the literature search, the authors (A.M.Č., B.M. and Z.B.M.) conceptualized the report in the form of a narrative review. Authors with high expertise in this field (B.M. and Z.B.M.) reviewed and revised the manuscript.

## Figures and Tables

**Table 1:** Hurley staging system [9].

**Hurley stage I**	single or multiple isolated abscesses without scars and sinus tracts (68% of patients)
**Hurley stage II**	recurrent abscesses with single or multiple scars and sinus tracts; widely separated lesions (28% of patients)
**Hurley stage III**	multiple lesions, extensive scars, and sinus tracts involving the entire region (4% of patients)

**Table 2:** International Hidradenitis Suppurativa Severity Score System (IHS4) [11].

**Mild HS**	3 or less than 3 points
**Moderate HS**	4–10 points
**Severe HS**	11 or more than 11 points
**IHS4 points = number of nodules × 1 + number of abscesses × 2 + number of draining fistulas × 4**	

Abbreviations: HS: Hidradenitis Suppurativa.

**Table 3:** Anti-TNF-α agents for HS treatment.

Biologic Drug	Structure	Studies	Dosage Regimen	Efficacy
ADALIMUMAB	Human recombinant IgG1 monoclonal antibody	Phase III RCT (PIONEER I) (*n* = 307) [64]	week 0–160 mg sc week 2–80 mg sc from week 4–40 mg sc weekly	41.8% of patients treated with ADA achieved HiSCR after week 12 vs. 26.0% of patients treated with placebo
		Phase III RCT (PIONEER II) (*n* = 326) [64]	week 0–160 mg sc week 2–80 mg sc from week 4–40 mg sc weekly	58.9% of patients treated with ADA achieved HiSCR after week 12 vs. 27.6% of patients treated with placebo
		Phase III OLE of PIONEER I and II (*n* = 88) [69]	every week–40 mg sc	62.5% of patients achieved HiSCR at week 36, and 52.3% of patients achieved HiSCR at week 168
		Retrospective real-life cohort study (*n* = 101) [70]	week 0–160 mg sc week 2–80 mg sc from week 4–40 mg sc weekly	77% of patients showed improving in HS-PGA
		Retrospective real-life cohort study (*n* = 389) [73]	week 0–160 mg sc week 2–80 mg sc from week 4–40 mg sc weekly	43.7% of patients achieved HiSCR at week 16, and 53.9% of patients achieved HiSCR at week 52
ADALIMUMAB BIOSIMILAR SB5	Human recombinant IgG1 monoclonal antibody	Retrospective observational study (*n* = 11) [78]	week 0–160 mg sc week 2–80 mg sc from week 4–40 mg sc weekly	54.5% of patients achieved HiSCR at week 36
INFLIXIMAB	Chimeric human/mouse IgG1 monoclonal antibody	Phase II RCT (*n* = 38) [80]	weeks 0, 2, 4, 6, 14, 22–5 mg/kg iv	26.7% of patients treated with IFX had 50% or greater decrease in HSSI vs. 5% of patients treated with placebo
		Prospective cohort study (*n* = 42) [83]	weeks 0, 2, 6–7.5 mg/kg iv from week 10–7.5 mg/kg or 10 mg/kg iv every 4 weeks	70.8% of patients treated with IFX 7.5 mg/kg achieved HS-PGA 0–2 at week 12; 50% of patients treated with IFX 10 mg/kg achieved HS-PGA 0–2 at week 12
INFLIXIMAB BIOSIMILAR (IFX-abda)	Chimeric human/mouse IgG1 monoclonal antibody	Retrospective cohort study (*n* = 34) [88]	weeks 0, 2, 6–10 mg/kg iv from week 10–10 mg/kg iv every 4/8 weeks	71% of patients treated with IFX-abda achieved HiSCR vs. 60% of patients treated with IFX
ETANERCEPT	Human recombinant TNF-receptor p75 Fc-IgG1 fusion protein	Phase II RCT (*n* = 20) [90]	every week–2 × 50 mg sc	There was no statistically significant difference in HS-PGA at weeks 12 or 24 between treatment and placebo groups
GOLIMUMAB	Human recombinant IgG1 monoclonal antibody	Case report [92] Case report [93]	every 4 weeks–50 mg sc; week 0–200 mg sc from week 4–100 mg sc every 4 weeks	HS-PGA deteriorated from 5 to 8; Remission of HS after 2 months
CERTOLIZUMAB PEGOL	Pegylated humanized monoclonal antigen- binding fragment (Fab’) of IgG	Case reports (*n* = 3) [95,96,97] Retrospective study (*n* = 2) [98]	every 2 weeks–400 mg sc; every 2 weeks–200 mg sc	Good disease control in 3 case reports; No efficacy

Abbreviations: ADA: adalimumab; DLQI: Dermatology Life Quality Index; HiSCR: Hidradenitis Suppurativa Clinical Response; HS-PGA: Hidradenitis Suppurativa–Physician Global Assessment; HSSI: the Hidradenitis Suppurativa Severity Index; IFX: infliximab; iv: intravenously; OLE: Open-Label Extension; RCT: Randomized Controlled Trial; sc: subcutaneously.

**Table 4:** Anti-IL-17 agents for HS treatment.

Biologic Drug	Structure	Studies	Dosage Regimen	Efficacy
SECUKINUMAB	Human IgG1κ monoclonal antibody	Open-label trial (*n* = 9) [100]	weeks 0, 1, 2, 3, 4–300 sc mg from week 8–300 mg sc every 4 weeks	78% of patients achieved HiSCR at week 24
		Open-label trial (*n* = 20) [101]	weeks 0, 1, 2, 3, 4–300 mg sc from week 6/8–300 mg sc every 2/4 weeks	70% of patients achieved HiSCR at week 24
		Retrospective cohort study (*n* = 20) [102]	weeks 0, 1, 2, 3, 4–300 mg sc from week 8–300 mg sc every 4 weeks	75% of patients achieved HiSCR by week 16
		Phase III RCTs (NCT03713619, NCT03713632, NCT04179175) [107,108,109]	Ongoing	
BRODALUMAB	Human IgG2 monoclonal antibody	Open-label trial (*n* = 10) [111]	weeks 0, 1, 2–210 mg sc from week 4–210 mg sc every 2 weeks	100% of patients achieved HiSCR
		Open-label trial (*n* = 10) [112]	every week–210 mg sc	100% of patients achieved HiSCR
BIMEKIZUMAB	Humanized IgG1κ monoclonal antibody	Phase II RCT (*n* = 90) [115]	week 0–640 mg sc from week 2–320 mg sc every 2 weeks	57.3% of patients achieved HiSCR at week 12 vs. 26.1% of patients treated with placebo
		Phase III RCTs (NCT04242446, NCT04242498, NCT04901195) [116,117,118]	Ongoing	
IXEKIZUMAB	Humanized IgG4κ monoclonal antibody	Case reports (*n* = 3) [120,121,122]	week 0–160 mg sc weeks 2, 4, 6, 8, 10, 12–80 mg sc from week 16–80 mg sc every 4 weeks	Good disease control
CJM112	Human IgG1κ monoclonal antibody	Phase II RCT (NCT02421172) (*n* = 66) [124]	weeks 0, 1, 2, 3, 4–300 sc mg from week 6–300 mg sc every 2 weeks	HS-PGA response rate with CJM112 32.5% vs. 12.5% with placebo

Abbreviations: HiSCR: Hidradenitis Suppurativa Clinical Response; HS-PGA: Hidradenitis Suppurativa–Physician Global Assessment; RCT: Randomized Controlled Trial; sc: subcutaneously.

**Table 5:** Anti-IL-12/23 agents for HS treatment.

Biologic Drug	Structure	Studies	Dosage Regimen	Efficacy
USTEKINUMAB	Human IgG1κ monoclonal antibody	Phase II open- label trial (*n* = 17) [126]	weeks 0, 4, 16, 28–45 mg sc if under 90 kg and 90 mg if over 90 kg	47% of patients achieved HiSCR at week 40
		Multicentre retrospective study (*n* = 14) [127]	week 0—iv infusion adjusted by weight (≤55 kg, 260 mg; 55–85 kg, 390 mg; ≥85 kg, 520 mg) from week 8–90 mg sc every 8 weeks	50% of patients achieved HiSCR at week 16
		Prospective study (*n* = 6) [128]	week 0—iv infusion adjusted by weight (≤55 kg, 260 mg; 55–85 kg, 390 mg; ≥85 kg, 520 mg) from week 8–90 mg sc every 8 weeks	50% of patients achieved HiSCR at week 12
		Case series (*n* = 10) [129]	every 8/12 weeks– 90 mg sc	70% of patients showed improvement in HS-PGA score
		Case series (*n* = 10) [130]	every 8 weeks–90 mg sc	90% of patients achieved HiSCR

Abbreviations: HiSCR: Hidradenitis Suppurativa Clinical Response; iv: intravenously; sc: subcutaneously.

**Table 6:** Anti-IL-23 agents for HS treatment.

Biologic Drug	Structure	Studies	Dosage Regimen	Efficacy
GUSELKUMAB	Human IgG1κ monoclonal antibody	A phase II RCT (NCT03628924) (*n* = 184) [138]	weeks 0, 4, 8, 12–200 mg sc; weeks 0, 4, 8–1200 mg iv week 12–200 mg sc	50.8% of participants achieved HiSCR at week 16; 45% of participants achieved HiSCR at week 16
		Case series (*n* = 4) [137]	week 0–100 mg sc from week 4–100 mg every 4 weeks	Variable results
		Case reports (*n* = 14) [132,133,134,135,136]	week 0–100 mg sc from week 4–100 mg every 8 weeks	Variable results
RISANKIZUMAB	Human IgG1κ monoclonal antibody	Case reports (*n* = 4) [141,142,143]	weeks 0, 4–150 mg sc from week 16–150 mg every 12 weeks	Positive results
		A phase II RCT (NCT03926169) (*n* = 243)	ongoing	
TILDRAKIZUMAB	Humanized IgG1κ monoclonal antibody	Case series (*n* = 4) [146,147]	weeks 0, 4–100 mg sc from week 8–200 mg every 4 weeks	100% of patients achieved HiSCR at week 8

Abbreviations: HiSCR: Hidradenitis Suppurativa Clinical Response; iv: intravenously; RCT: Randomized Controlled Trial; sc: subcutaneously.

**Table 7:** Anti-IL-1 agents for HS treatment.

Biologic Drug	Structure	Studies	Dosage Regimen	Efficacy
ANAKINRA	Human recombinant IL-1 receptor (IL-1R) monoclonal antibody	Phase II RCT (*n* = 20) [150]	100 mg sc g.d./12 weeks	78% of patients treated with anakinra achieved HiSCR vs. 30% of patients treated with placebo
		Case series (*n* = 6) [151]	100 mg sc g.d./8 weeks	A rebound of the disease 8 weeks after the end of the treatment
		Case reports (*n* = 4) [92,153,154]	100 mg sc g.d.	Failure
BERMEKIMAB	Human recombinant IgG1κ monoclonal antibody	A phase II RCT (NCT02643654) (*n* = 20) [156]	every 2 weeks–7.5 mg/kg iv	60% of patients treated with bermekimab achieved HiSCR vs. 10% of patients treated with placebo
		OLE of NCT02643654 (*n* = 8) [157]	every 2 weeks–7.5 mg/kg iv	75% of patients achieved HiSCR at week 12
		Open-label trial (*n* = 42) [158]	every week–400 mg sc	61% of patients naive to anti-TNF therapy and 63% having failed anti-TNF therapy achieved HiSCR at week 12
		Phase II RCT (NCT04988308) (*n* = 290) [159]	ongoing	
CANAKINUMAB	Human recombinant IgG1κ monoclonal antibody	Case reports (*n* = 6) [161,162,163,164]	every week/4 weeks/8 weeks–150 mg sc	Variable results

Abbreviations: HiSCR: Hidradenitis Suppurativa Clinical Response; iv: intravenously; OLE: Open-Label Extension; RCT: Randomized Controlled Trial; sc: subcutaneously.
